# Tofacitinib for elderly onset hemophagocytic lymphohistiocytosis with gene mutations: a case report

**DOI:** 10.1002/mco2.538

**Published:** 2024-04-13

**Authors:** Tingting Liu, Zhi‐Peng Cheng, Yu Hu, Liang V. Tang

**Affiliations:** ^1^ Institute of Hematology, Union Hospital, Tongji Medical College, Huazhong University of Science and Technology Wuhan China

Dear Editor,

An 85‐year‐old man presented with a 2‐month history of recurrent fever, with a peak temperature of 38.5–39°C, accompanied by chills, cough, and fatigue. The patient received symptomatic anti‐infection treatment in a local hospital, but he continued to experience stubborn fever. On admission, the patient showed a pancytopenia. Subsequent investigations revealed an elevated ferritin level and a decrease in natural killer cell function. Bone marrow showed a hemophagocyte of 0.5% (Figure 1A,B). These data were consistent with the diagnosis of hemophagocytic lymphohistiocytosis (HLH).^1^

**FIGURE 1:**
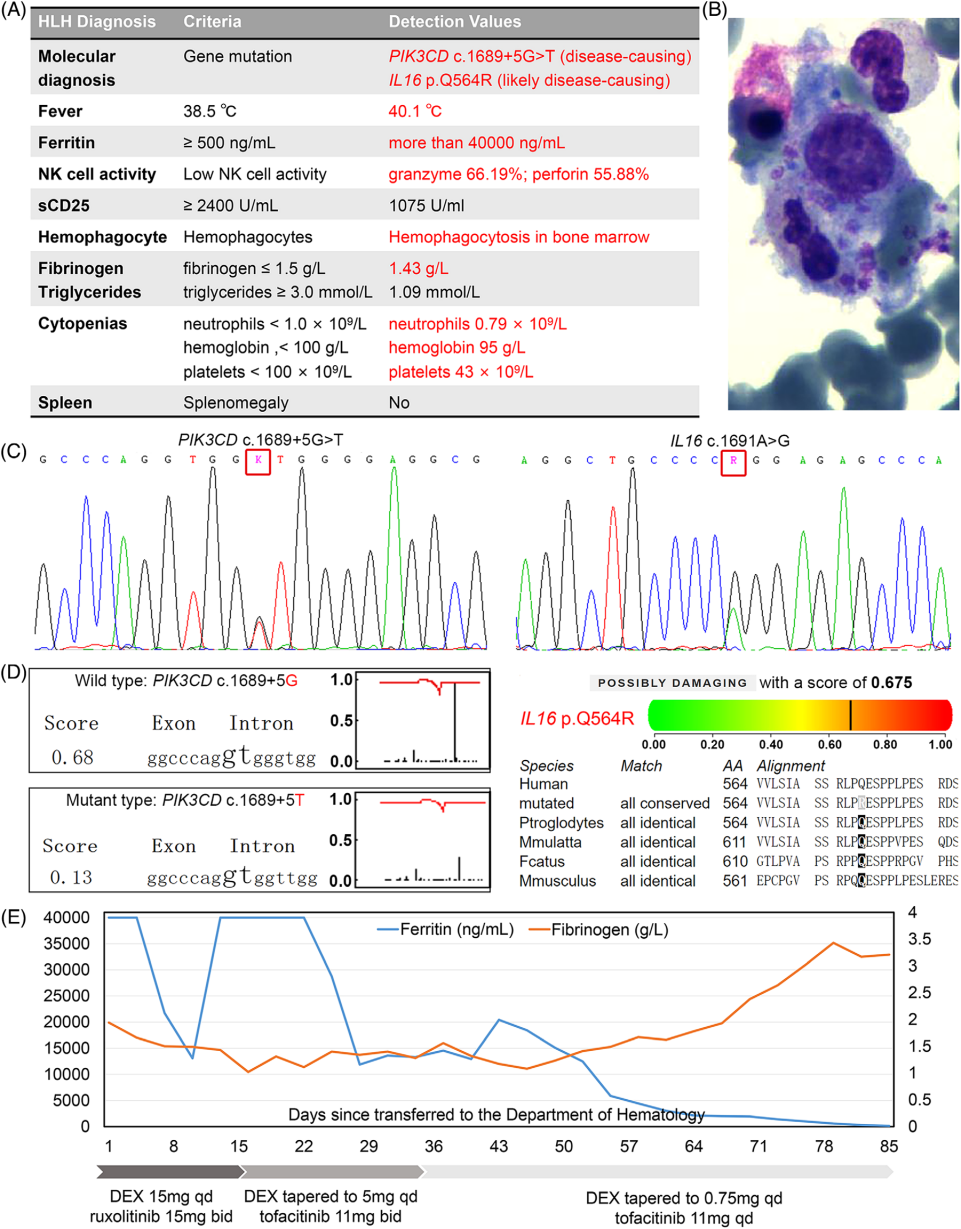
Diagnostic evidence and clinical course (A) The diagnostic criteria for HLH and the detection values of the patient. NK cell, natural killer cell; sCD25, soluble CD25 or soluble interleukin‐2 receptor; (B) Hemophagocytosis in the patients’ bone marrow: a hemophagocyte is engulfing a normal blood cell (1000 × objective); (C) Chromatograms of the *PIK3CD* c.1689+5G > T mutation and the *IL16* c.1691A > G mutation: validated by Sanger sequencing; (D) Pathogenicity and functional consequence of the *PIK3CD* c.1689+5G > T mutation: this mutation located at one of the key donor splice sites of exon 13 in *PIK3CD*. It was damaging because the splicing efficacy of mRNA was seriously impacted, predicted by both the BDGP (https://www.fruitfly.org/seq_tools/splice.html) and the NetGene2 (https://services.healthtech.dtu.dk/services/NetGene2‐2.42); Pathogenicity and functional consequence of the *IL16* c.1691A > G (p.Q564R) mutation: the missense mutation in the coding sequences was predicted to be possibly damaging by both the multiple‐sequence‐alignment and the PolyPhen‐2 (http://genetics.bwh.harvard.edu/ggi/cgi‐bin/ggi2.cgi); (E) Clinical course of the HLH patient. The upper detection limit of serum ferritin level in the Department of Laboratory in our hospital is 40,000 ng/mL (normal range, 21.8–275); If the patient's fibrinogen level drops to less than 1.3 g/L, human fibrinogen infusion (1 g) would be prescribed; DEX, dexamethasone.

The underlying etiology of HLH such as active infection, tumor, and rheumatic diseases had been excluded step by step (Table S1). Whole exome sequencing was performed (Figure S1), revealing two novel compound heterozygous damaging mutations: a splice site mutation in *PIK3CD* (c.1689+5G > T) and a missense mutation in *IL16* (c.1691A > G or p.Q564R) (Figure 1C,D). The former was a classic HLH‐related gene. The latter might also be potentially associated with HLH, which requires further validation. According to the American College of Medical Genetics and Genomics standards and guidelines, both of the two gene mutations were classified as pathogenic. Mutations in the *PIK3CD* gene were recently described as an inborn error of immunity associated with recurrent infections and lymphoproliferative diseases. Even monoallelic mutations can affect the regulation of the PI3K signaling pathway, resulting in abnormal function of T cells, and overproduction of inflammatory factors, leading to the development of HLH.^2^ Meanwhile, IL16 is one of the new candidate genes contributing to the pathogenesis of HLH. Mutations in *IL16* may affect the transduction process of inflammatory signals and the immune response to pathogens.^3^ Therefore, the patient met the molecular criteria for primary HLH.

Because of the advancing age, the patient refused to use cytotoxic drugs such as etoposide. Because of the lack of medication experience in the elderly, he also refused to use emapalumab. The patient received dexamethasone in combination with ruxolitinib 15 mg bid. The patient's ferritin level temporarily decreased to 13,063.4 ng/mL but quickly rebounded to over the upper detection limit, 40,000 ng/mL (Figure 1E). Additionally, the patient's peak temperature increased to 40.1°C, and there was a further decline in the patient's blood cell counts and fibrinogen level. In order to control HLH, we tried another JAK inhibitor that targeted the inflammatory cytokine storm: tofacitinib 11 mg bid. The patient responded well with marked improvement in body temperature, pancytopenia, and hyperferritinemia. Three weeks later, the dosage of tofacitinib was adjusted to 11 mg once daily, in order to avoid opportunistic infections. He continued to take oral tofacitinib after discharge and gradually achieved a complete remission (Figure S2).

HLH is a serious, life‐threatening complex disorder. It is characterized by an excessive inflammatory response, wherein multiple inflammatory factors such as interferon‐γ, bind with receptors through Janus kinases (JAK)1‐3 and thus activate the downstream target genes.^4^ JAK inhibitors can block the JAK‐STAT pathway, inhibit the activity of JAK, and suppress the secretion of inflammatory factors. Therefore, JAK becomes a target for the treatment of HLH. Currently, among several JAK inhibitors, only ruxolitinib has been focused on for the management of HLH. Ruxolitinib is primarily approved for the treatment of myelofibrosis and myeloproliferative neoplasms. It can also play a role in controlling HLH in experimental models and clinical trials.^5^, ^6^, ^7^, ^8^, ^9^ However, in this case, the patient showed poor response after 2‐week therapy with ruxolitinib. We speculate that ruxolitinib mainly targets bone marrow, and we may choose JAK inhibitors that mainly focus on the immune system. Tofacitinib is another oral JAK inhibitor that has a more powerful immunosuppressive effect but lesser myelosuppression. It is primarily utilized to treat rheumatoid arthritis, ulcerative colitis, and other inflammatory diseases in clinical settings.^10^ As expected, tofacitinib demonstrated therapeutic benefit in this case.

In summary, this 85‐year‐old man is to date the oldest patient affected with HLH‐associated mutations reported worldwide, according to the literature. This case serves as a reminder for clinicians to consider the possibility of primary HLH or a genetic background, even in elderly patients. Screening for underlying gene mutations is helpful in elderly adults with unexplained HLH. Moreover, this is the first report of using tofacitinib for successfully managing HLH, indicating a promising agent. The safety and efficacy of tofacitinib should be further validated in large clinical trials.

## AUTHOR CONTRIBUTIONS

L.V.T. and T.T.L. wrote the initial draft. L.V.T., Z.P.C., and H.Y. revised the manuscript. L.V.T., T.T.L., Z.P.C., and Y.H. read and approved the final manuscript.

## CONFLICT OF INTEREST STATEMENT

The authors declare no conflict of interest.

## FUNDING INFORMATION

This case study was supported by the Chang Jiang Scholars Program (No. 2022161), the National Natural Science Foundation of China (No. 82170131), and the Young Top‐notch Talent Cultivation Program of Hubei Province (No. 202117).

## ETHICS STATEMENT

This study was approved by the Ethics Committee of the Union Hospital affiliated with Huazhong University of Science and Technology (No.2022‐0370). Written informed consent was provided by the patient to publish this study.

## Supporting information

Supporting information

## Data Availability

The data are available from the corresponding author upon reasonable request.
