# Supplementary material for: Tofacitinib for elderly onset hemophagocytic lymphohistiocytosis with gene mutations: a case report
Source: MedComm (2020). 2024 Apr 13;5(4):e538. doi: 10.1002/mco2.538 (PMC11016134; doi:10.1002/mco2.538)
Supplement: Supplementary file 1 — Supporting information [file MCO2-5-e538-s001.docx]

**Supplementary Data**

**Tofacitinib for Elderly Onset Hemophagocytic Lymphohistiocytosis with Gene Mutations: A Case Report**

Tingting Liu^a^, Zhi-Peng Cheng^a^, Yu Hu^a^, Liang V. Tang^a,*^

^a^ Institute of Hematology, Union Hospital, Tongji Medical College, Huazhong University of Science and Technology, Wuhan, PR China

^*^Correspondence: [lancet_tang@hust.edu.cn](mailto:lancet_tang@hust.edu.cn)

**Figure S1. Whole Exome Sequencing**


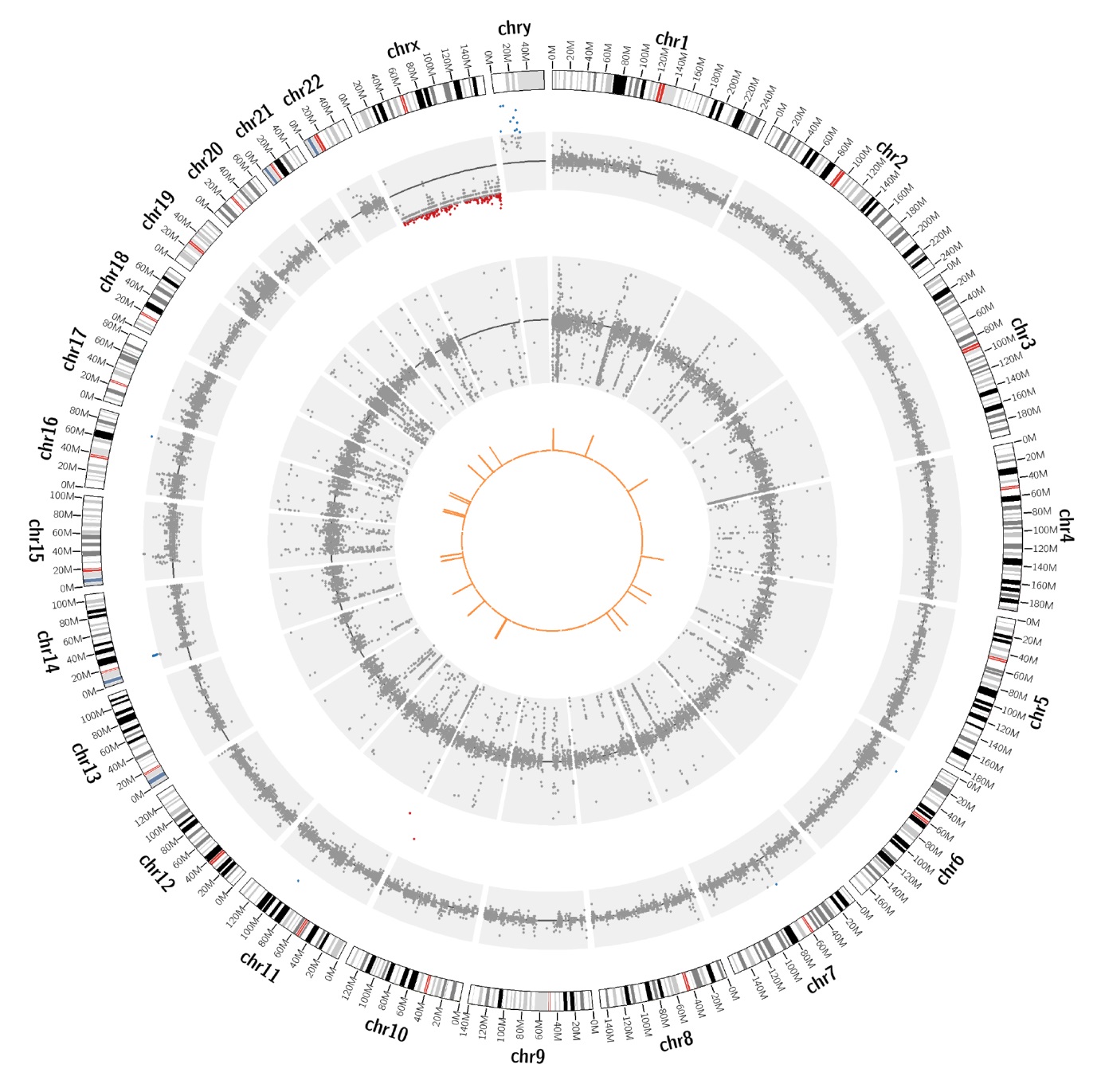


Genomic DNA was extracted from the elderly HLH patient’s peripheral white blood cells as well as pharyngeal swab samples, respectively. Whole exome sequencing was performed on an Illumina (NextSeq550DX/NovaSeq6000). It covered the whole exon regions of around 19,500 genes, and detected mutations including single nucleotide variations, small insertions, small deletions, and copy number variations. The average depth of re-sequencing was 843.3 ×, and the Q30 ratio was 94.0 %. According to the <Standards and Guidelines for the Interpretation and Reporting of Sequence Variants in Cancer>, somatic mutations are divided into four categories according to their clinical significance: Type I, mutations with definite clinical significance; Class II, of potentially clinically significance; Category III, variations of uncertain clinical significance; Category IV, harmless or potentially harmless mutation. According to the American College of Medical Genetics and Genomics standards and guidelines, germline variations are divided into 5 grades: pathogenic variation, possible pathogenic variation, variation of unknown clinical significance, possible benign variation, and benign variation. Germline mutations are validated by Sanger sequencing using an independent DNA sample.

**Figure S2. Dynamic Changes of Laboratory Data**


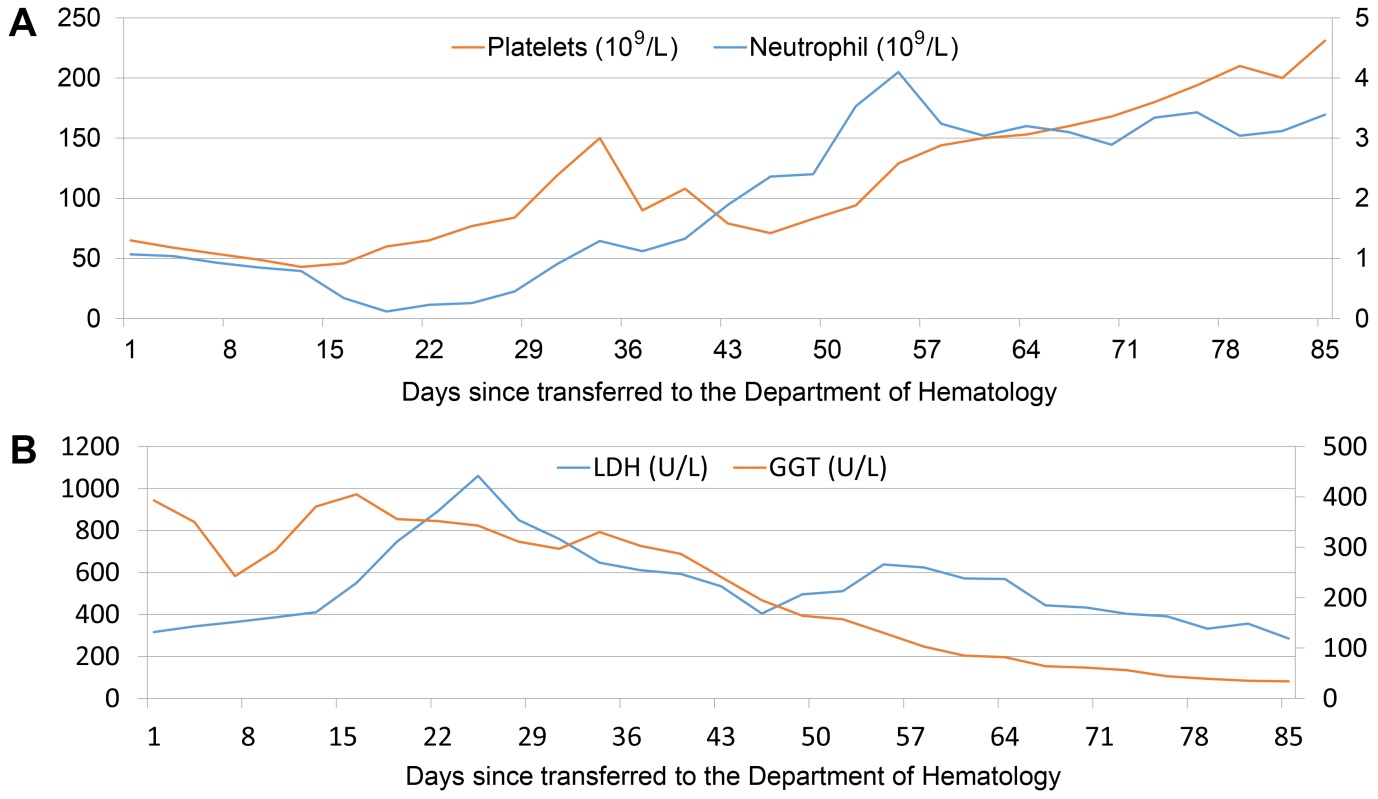


Normal range for platelet: 125~350 × 10^9^/L; Normal range for neutrophil: 1.8~6.3 × 10^9^/L; LDH: lactic dehydrogenase, 109~245 U/L; GGT: γ-gluyamyl transferase, 7~32 U/L

**Table S1. Screening for the Etiologies of HLH**

| **Tests** | **Results** |
| --- | --- |
| Epstein-Barr virus DNA (whole blood) | Negative |
| Epstein-Barr virus DNA (plasma) | Negative |
| Cytomegalovirus DNA (whole blood) | Negative |
| Cytomegalovirus DNA (plasma) | Negative |
| New Bunya Virus RNA | Negative |
| Tuberculosis T-spot | Negative |
| HIV antibody | Negative |
| Metagenome high-throughput sequencing  (10989 species of bacteria, including 196 species of mycobacteria and 159 species of mycoplasma/ Chlamydia/rickettsia; 5050 types of virus; 1179 species of fungus; 282 kinds of parasite) | Negative |
| Anti-nuclear antibodies spectrum | Negative |
| Rheumatoid factors | Negative |
| Anti-neutrophilcytoplastic antibodies | Negative |
| Serum tumor markers | Negative |
| Bone marrow biopsy | No morphological, immunophenotypic, chromosomal, or clonal abnormalities |
| PET-CT scan | The number of bilateral cervical, mediastinal and inguinal lymph nodes increased, the morphology was normal, and the metabolism was not high, which was considered as non-specific inflammatory reaction. |

HIV: human immunodeficiency virus; PET: positron emission tomography
